# Do Giant Pandas Prefer Steeper Habitats? A Case Study on Panda Spatial Utilization in the Qinling Mountains, China

**DOI:** 10.1002/ece3.71074

**Published:** 2025-03-05

**Authors:** Chun Yang, Wanyu Wen, Yuhang Wang, Zhijiao Yang, Changcai Li, Simeng Yang, Xinyu Li, Minghao Gong

**Affiliations:** ^1^ Institute of Wetland Research Chinese Academy of Forestry Beijing China; ^2^ Institute of Ecological Conservation and Restoration Chinese Academy of Forestry Beijing China

**Keywords:** giant panda, habitat, slope, spatial utilization

## Abstract

Animal‐trace data from the Third and the Fourth National Giant Panda Survey in the four reserves in the Shaanxi Qinling Mountains (Laoxiancheng, Foping, Changqing, and Huangbaiyuan) suggested that giant pandas unexpectedly have a lower occurrence rate in gentle locations. To explore the cause of this apparently counterintuitive preference, we used spatial and data analysis tools to analyze the spatial composition of the daily activity zones, the relative quantity of traces, the trace density, and the slope supply of the reserves. We found that the slope composition around gentle slopes and steep slopes is similar, with more traces clustered around gentle slopes. The area of the reserves with a 5°–15° slope is very small, and the density of traces is negatively correlated with the slope. So the slope distribution of giant panda traces is highly correlated with environmental supply. A reduced supply of suitable habitats leads to diminished availability, ultimately resulting in a narrower distribution range for giant pandas.In addition, combining the spatial supply situation, the use of trace density can more accurately reflect animal habitat selection preferences, which is more in line with the optimal foraging theory and animal habits. This study provides insights that should benefit future assessment and restoration of their habitats.

## Graphical abstract

Traces of Qinling giant pandas are more frequent on steep slopes, and this unusual pattern is attributable to lower availability of gentle slopes. Compared to steep slopes, giant pandas prefer flat land around gentle slopes. The density of traces in gentle land is higher than in steeper land, so panda distribution followed optimal foraging when preferred sites were available.

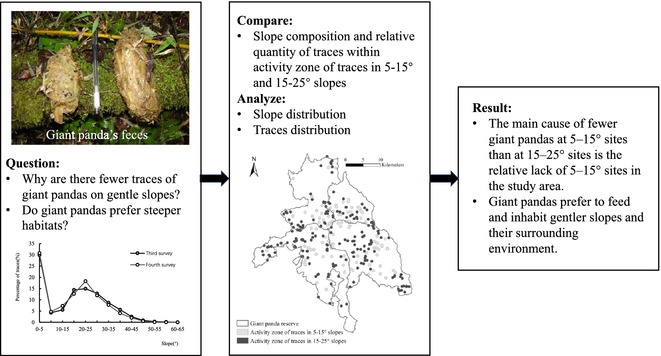

## 1 Introduction

The selection of a suitable habitat yields major fitness rewards for animals in terms of increased resource (food or mating) availability and safety, while minimizing energy expended to obtain these benefits (O'Brien et al. 1989; Steven and Lawrence 1990; Carla et al. 2021). Among the characteristics that influence habitat selection, the slope of the landscape appears to have a considerable impact (Liu et al. 2005; Kang 2015; Hull et al. 2016; Gong et al. 2017a). Several tree‐climbing primate species, such as Francois' langur ( *Trachypithecus francoisi* ) and black‐and‐white snub‐nosed monkeys ( *Rhinopithecus bieti* ), prefer steep slopes (> 20°) (Li et al. 2006; Han et al. 2023), whereas exclusively land‐dwelling species—giant pandas ( *Ailuropoda melanoleuca* ), wild boars (*Sus scrofa*), and Mongolian antelopes ( *Procapra gutturosa* )—prefer flat areas (< 20°) because rugged terrain is not conducive to the movement of the latter species (Tong 2012; Han 2022). These preferences likely reflect a combination of selective pressures related to refuge from predators and ease of travel.

To maintain a balance between energy expenditure and intake, giant pandas prefer to forage in bamboo forests with gentle slopes. Such areas have several advantages. First, they minimize the high‐intensity activity needed to navigate rugged terrain (Jiang 2004; Sun 2019). Second, flatter surfaces allow pandas to consume bamboo while seated, their preferred posture for feeding (Yang et al. 2006; Kang et al. 2017). Third, flat ground tends to have a thick soil texture with hydrothermal conditions that are conducive to bamboo growth (Dang et al. 2012). Panda preferences thus support the optimal foraging theory (OFT), wherein animals are posited to minimize investment and maximize returns when foraging (Shang 2014). However, several studies seem to indicate that this preference for gentle slopes does not always hold. For example, the number of giant panda traces distributed at 0°–20° in Foping and Changqing nature reserves was significantly lower than that distributed at 20°–40° (Liu and Jin 2008). These phenomena seem to conflict with the OFT, leading to misconceptions regarding the behavior of giant pandas and questioning the suitability classification of existing habitats.

Spatial utilization characteristics are an important basis for classifying and evaluating habitat suitability. Biologically relevant classification is essential for accurately evaluating the value of regions under consideration for protection. Currently, the majority of research on slope effects on animal locomotion and habitat selection primarily focuses on the frequency of animal traces in different slope intervals (Shen et al. 2002; Zhu 2016; Sun et al. 2022; Chen et al. 2022). Although such data can provide insights into animal preferences, spatial utilization depends on a combination of biological characteristics and environmental conditions. If the availability of sloped terrain is not considered, foraging patterns may appear to contradict the OFT and lead to unnecessary controversy, while also negatively impacting subsequent habitat evaluation, spatial management, and maintenance strategies (Li 2006).

To address this problem, we conducted an in‐depth analysis of panda spatial use in four protected areas of the Qinling Mountains, as well as their preference for slope steepness. Our specific objectives were to: (1) explore the causes of lower giant panda distribution at gentle slope than at steep slope; (2) clarify terrain usage characteristics of giant pandas and factors that restrict said usage; (3) verify the applicability of OFT to giant pandas. Our findings would improve our understanding of panda behavioral ecology, improve the scientific validity of results from habitat suitability evaluations, and guide the management of giant panda habitats.

## 2 Methods

### 2.1 Study Site

The giant panda is endemic to China and is currently distributed in the forests of six mountain ranges, including the Qinling, Minshan, and Qionglai Mountains (Hu 2000). The fourth giant panda survey in 2012 counted approximately 345 wild giant pandas (excluding cubs under 1 year old) in the Shaanxi Qinling Mountains, accounting for 18.5% of all wild giant pandas in China. A population density of 0.096 pandas/km^2^ makes Qinling the habitat with the highest wild panda density in the country (State Forestry Administration 2014; Nan et al. 2021). According to the Third National Giant Panda Survey in 2000, the pandas are concentrated in the Xinglong Mountains, a Qinling branch at the junction of Foping, Yangxian, Zhouzhi, and Taibai counties (Li 2006; China's State Forestry Administration 2006). So, I chose this area as my study site.

**FIGURE 1:**
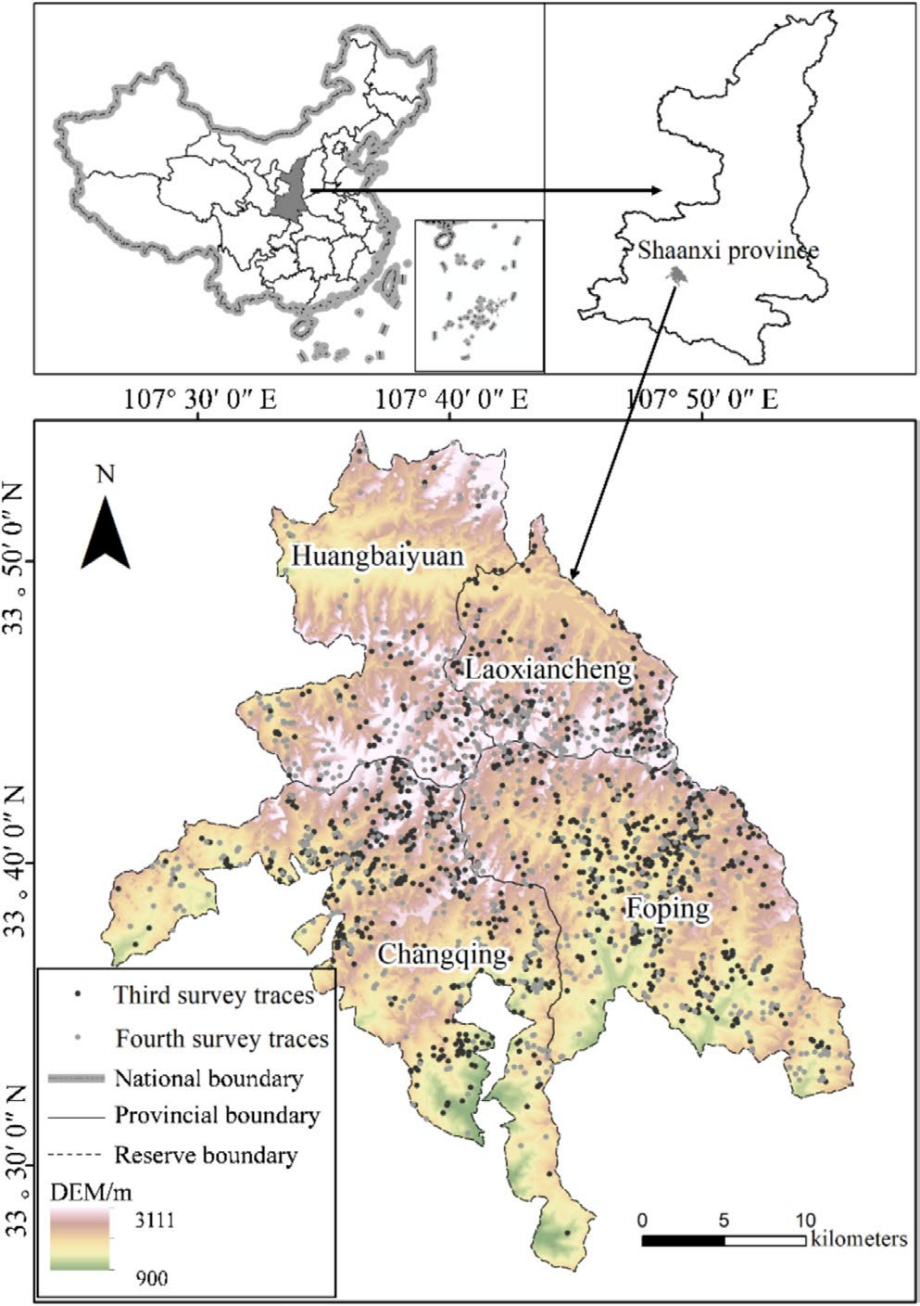
Map of the study area, marking out traces of giant pandas in the third and fourth national surveys.

The study site (Figure 1; 107°17′–107°55′ E and 33°19′–33°54′ N) encompasses four national nature reserves in Shaanxi: Laoxiancheng, Foping, Changqing, and Huangbaiyuan. The fourth national giant panda survey found 188 giant pandas in the four reserves, accounting for 54.5% of all Qinling pandas (China's State Forestry Administration 2014). The average slope in the region is 21.68°, with a range of 0°–69.30°. The maximum altitude is 3111 m in the northern part of the Huangbaiyuan Reserve, and the minimum altitude is 900 m in the southern part of the Changqing Reserve. The mountainous climate transitions from the northern subtropical zone to the warm temperate zone. The region is a part of the Han River system, a tributary of the Yangtze River.

### 2.2 Ethical Note

While foraging, giant pandas leave behind traces of habitat use, including feces, nests, sleeping places, and footprints. Utilizing the spline method to track the trail of pandas is a primary approach for researchers to determine the survival situation of pandas (Wen et al. 2009). Spline monitoring provides a comprehensive understanding of the spatial utilization of target species in an area and records species entities as well as trace information such as footprints, food tracks, and hair, with minimal gaps in data. It is an important basis for estimating population size, distribution, and density, without causing any harm to animals or the environment (Wang et al. 2024). Therefore, our research was non‐invasive observation; no animals were harmed.

### 2.3 Data Sources

Data on giant panda traces were obtained from the Third National Giant Panda Survey (TNGPS) from 1999 to 2003 (hereafter “the third survey”) and the Fourth National Giant Panda Survey (FNGPS) from 2011 to 2014 (hereafter “the fourth survey”), both conducted by the National Forestry Administration (State Forestry Administration 2006, 2014). These two surveys recorded 1810 and 1854 traces with longitude and latitude, respectively, and were collected within the known range of Qinling pandas at a frequency of one transect per 200 ha (Gong et al. 2017b). The Digital Elevation Model and terrain data were downloaded from Geospatial Data Cloud (http://www.gscloud.cn/). The digital elevation model was GDEMV2 with 30 m resolution. The administrative divisions and boundaries of panda protection areas were provided by the official authorities of each national nature reserves.

### 2.4 Data Analysis

#### 2.4.1 Data Pretreatment

Survey outcomes indicated that giant pandas in the study area used a slope range of 0°–60°. To obtain a more granular picture of slope utilization, slope span was set to 5° for statistical analysis and divided into 12 slope intervals. According to the number of traces in each slope interval, the anomaly interval was selected as the research group: GS (gentle slope), and the adjacent steeper slope interval was selected as the comparison group: SS (steep slope). The data from the third and fourth surveys are used as two repetitions. A 400‐m activity zone for both groups was delineated based on the average daily movement range of giant pandas being 411 m in diameter (Pan et al. 2001). The activity zones of GS and SS are respectively referred to as AZG and AZS.

#### 2.4.2 Parameters Calculation

Patches containing each slope interval were extracted from the activity zone, and their area percentage was calculated to determine whether the two activity zones differed in slope composition.

Calculate the relative quantity to reflect the aggregation degree of giant pandas in two activity zones, and determine which slope environment giant pandas are more inclined to center on and move around. The relative quantity is the ratio of the following two proportions: (1) the proportion of traces in each slope interval in activity zones to traces in each slope interval in the study area, and (2) the proportion of GS and SS to all trace points.

The trace density (TD, pcs/km^2^) of each slope interval was calculated based on the following formula:

(1) $${\mathrm{TD}}_{i}=\frac{N_{i}}{A_{i}}$$

where *i* represents the slope interval, *N*_*i*_ represents the number of trace points in the *i*th interval, and *A*_*i*_ represents the area of the *i*th interval.

All data were converted to GIS format based on the UTM WGS 84 coordinate system and analyzed in ArcGIS 10.7 (Esri, Redlands, CA, USA). The paired samples *t*‐test and Pearson's correlation analysis in SPSS (version 18.0; SPSS Inc., Chicago, IL, USA) were employed to examine the correlations among the parameter.

## 3 Results

### 3.1 Number of Giant Panda Traces in Each Slope Interval

According to the OFT and the characteristics of giant pandas, the traces number should decrease with increasing slope. However, the data from Table 1 show that the traces number in the 5°–10° and 10°–15° slopes is far less than in the 15°–20° and 20°–25° slopes in two surveys. The slope distribution characteristics of traces of giant pandas did not differ between the third and fourth surveys (*p >* 0.05, Table 1). This means that the slope utilization of the giant panda has not changed in the past decade. Does this indicate that giant pandas prefer steeper slopes (15°–25°) compared to gentle slopes (5°–15°)? Regarding this question, we selected the anomaly interval (5°–15°) as the GS and the adjacent steeper slope interval (15°–25°) as the SS for analysis.

**TABLE 1:** Distribution of giant panda traces in each slope interval during the two surveys.

Slope intervals (°)	Third survey		Fourth survey	
	Trace number (pcs)	Proportion (%)	Trace number (pcs)	Proportion (%)
0–5	378	29.88	358	30.76
5–10	54	4.27	55	4.73
10–15	70	5.53	85	7.30
15–20	182	14.39	147	12.63
20–25	189	14.94	213	18.30
25–30	162	12.81	139	11.94
30–35	110	8.7	90	7.73
35–40	71	5.61	47	4.04
40–45	32	2.53	23	1.98
45–50	11	0.87	6	0.52
50–55	4	0.32	1	0.09
55–60	2	0.16	0	0.00
Total	1265	100	1164	100

### 3.2 Slope Composition Within the Activity Zones

Through the paired sample *t*‐test, it was found that there is no significant difference between the slope composition of the two activity zones(*p >* 0.05, Table 2). This result rules out the possibility that the difference in the surrounding space of the two sets of traces may affect the giant panda's choice of slope. The diversity in geomorphic characteristics ensures that giant pandas have a variety of habitat options. Data showed that 5°–15° slopes took up a smaller area than 15°–25° slopes in the AZG (13.27% < 32.56%, Table 2). These findings indicate that even if the supply of 15°–25° areas is greater than that of 5°–15° areas, giant pandas will prioritize the latter. Therefore, it can be considered that giant pandas did not avoid the 5°–15° environment.

**TABLE 2:** Slope composition around the two groups.

Slope intervals (°)	Slope composition (%)	
	AZG	AZS
0–5	20.92 (0.58)	16.64 (1.07)
5–10	4.19 (0.21)	3.46 (0.02)
10–15	9.08 (1.75)	5.66 (0.05)
15–20	15.94 (0.08)	14.55 (0.94)
20–25	16.62 (1.13)	20.59 (0.06)
25–30	15.21 (0.16)	17.36 (0.64)
30–35	8.83 (0.25)	11.09 (0.18)
35–40	5.22 (0.84)	6.06 (0.37)
40–45	2.67 (0.72)	3.16 (0.25)
45–50	0.89 (0.35)	1.05 (0.06)
50–55	0.30 (0.11)	0.29 (0.01)
55–60	0.12 (0.04)	0.10 (0.00)

*Note:* In parentheses are standard deviations. AZG, activity zone of traces in 5°–15° slopes; AZS, activity zone of traces in 15°–25° slopes.

### 3.3 Distribution of Panda Traces Within the Activity Zones

To further investigate which slope giant pandas prefer to gather around, we extracted traces in AZG and AZS for comparison. In the third survey, the traces proportion of GS and SS is 9.8% and 29.3%, respectively. In the fourth survey, there are 12% and 30.9%, respectively (Table 1). These numbers are the denominator of relative quantities. Then, by combining the proportion of each slope traces in the activity zone, we found that the relative quantity of traces in AZG is greater than that in AZS (Table 3). There is a significant difference in the relative quantity between the two groups after the paired samples *t*‐test with *p* = 0.009 < 0.05. This outcome shows that under similar environmental conditions in the two activity zones, the aggregation of giant panda traces around gentle slopes (5°–15°) was significantly higher than around steep slopes (15°–25°), indicating that giant pandas mainly inhabit the gentle slopes as their habitat center.

**TABLE 3:** Relative quantity of traces in AZG and AZS.

Slope intervals (°)	Traces number				Relative quantity	
	AZG (Third survey)	AZS (Third survey)	AZG (Fourth survey)	AZS (Fourth survey)	AZG	AZS
0–5	76	66	47	53	1.57 (0.48)	0.54 (0.06)
25–30	19	48	20	23	1.20 (0.00)	0.77 (0.24)
30–35	17	30	6	12	1.07 (0.51)	0.68 (0.25)
35–40	13	13	1	10	1.02 (0.85)	0.66 (0.03)
40–45	4	6	0	3	0.64 (0.64)	0.53 (0.11)
45–50	2	4	1	3	1.62 (0.23)	1.43 (0.19)
50–55	1	1	0	0	1.28 (1.28)	0.43 (0.43)
55–60	0	0	0	0	—	—
Mean					1.20 (0.57)	0.72 (0.12)

*Note:* In parentheses are standard deviations. AZG, activity zone of traces in 5°–15° slopes; AZS, activity zone of traces in 15°–25° slopes.

### 3.4 Slope Supply Situation and Traces Density in the Study Area

The slope 5°–15° sites were far smaller in area than the slope 15°–25° sites (8.87% < 27.38%, Table 4). We did not observe a significant difference between the proportion of areas and the proportion of panda traces within each slope interval (*p* > 0.05), indicating that the distribution of giant pandas was consistent with the supply of slopes. Pearson's correlation analysis shows a high negative correlation between the slope area and the density of giant panda traces (*r* = −0.952, *p* < 0.01). The combination of the number of traces with the area of each slope interval reflects a result that aligns with the OFT. As the slope increases, the density of traces gradually decreases.

**TABLE 4:** Area and density of panda traces within each slope interval.

Slope intervals (°)	Area (km^2^)	Proportion of total site area (%)	Density of panda traces (pcs/km^2^)
0–5	177.69	18.88	2.07 (0.06)
5–10	32.51	3.46	1.68 (0.02)
10–15	50.88	5.41	1.53 (0.15)
15–20	104.79	11.14	1.57 (0.17)
20–25	152.78	16.24	1.32 (0.08)
25–30	155.28	16.5	0.97 (0.07)
30–35	122.15	12.98	0.82 (0.08)
35–40	79.53	8.45	0.74 (0.15)
40–45	42.48	4.51	0.65 (0.11)
45–50	16.17	1.72	0.53 (0.16)
50–55	4.88	0.52	0.51 (0.31)
55–60	1.55	0.16	0.65 (0.65)
Total	941.02	100	13.02 (1.5)

*Note:* In parentheses are the standard deviations.

## 4 Discussion

Our study used the total trace distribution pattern as an indicator of the spatial preferences of the giant pandas. We specifically investigated traces distributed in and around 5°–15° and 15°–25° slope angles. These two groups of panda traces allowed us to determine preferences for gentle versus steep slope environments. A major conclusion of our study is that compared with 15°–25°, Qinling giant pandas actually tend to be more active on slopes of 5°–15° and their vicinity, as evidenced by the relative quantity and density of traces. The main cause of fewer giant panda traces at slope 5°–15° sites than at slope 15°–25° sites is the relative lack of 5°–15° sites in the study area. An insufficient supply forces pandas to go elsewhere.

So if a preferred environment is unavailable, giant pandas will move to relatively unsuitable environments despite their preferences. Apart from the fundamental reason of insufficient supply, the reason why the more suitable environment is unavailable may also be that it is occupied and used by sympatric animals. For example, the Qinling takin ( *Budorcas taxicolor bedfordi* ), which overlaps with the habitat space of giant pandas, also prefers to move on slopes of 10°–30° (Ding and Liu 2022; Qi and Gong 2023). Meanwhile, human utilization of gentle slopes can also cause giant pandas to avoid them (Yang et al. 2024). Similar phenomena have also been observed in other species. For example, red‐crowned cranes ( *Grus japonensis* ) will choose to reside on farmland when their traditional habitats decrease and human interference increases; however, farmland is not necessarily the preferred habitat for this species (Wu et al. 2013; Li et al. 2013; Wang et al. 2019; Wang et al. 2020). Our study is one of the first to directly assess the impact of spatial accessibility. Spatial factors, such as terrain, altitude, and slope, are less variable than food, with potentially more permanent effects on foraging behavior. To gain comprehensive insights into animal movements, spatial accessibility should be an essential component of future research on terrestrial animals, particularly large species, such as giant pandas (Bai et al. 2020).

Our research is also of great significance for further enhancing the scientific validity of habitat suitability assessment. The 5°–15° slope would be classified as suboptimal if conclusions were drawn based only on the raw frequency of panda traces. However, when we accounted for the space area, 5°–15° became classified as the most suitable slope angle. In other words, we considered habitat availability in addition to animal distribution to obtain a more accurate view of panda foraging preferences and habitat suitability.

During the line monitoring, although staff encountered live giant pandas by chance, many parts of the protected areas are inaccessible to humans due to the high altitude and steep terrain of the Qinling Mountains. Inevitably, some traces of giant pandas have not been discovered yet. Therefore, the actual habitat range of giant pandas could potentially exceed the current findings, necessitating the need for collecting more comprehensive data for more accurate conclusions in the future.

## 5 Conclusions

Our study is based on the OFT and the behavioral ecology of giant pandas in the Qinling Mountains. The relative quantity of traces in two groups of daily activity zones is used to represent the degree of aggregation of giant pandas on gentle and steep slopes. The density of traces is used to reflect the intensity of giant panda slope utilization. The following conclusions are drawn: (1) The slope distribution of giant panda traces is highly correlated with environmental supply. The less supply, the lower the availability, and the less distribution of giant pandas; (2) The utilization intensity of giant pandas decreases with slope increasing; (3) Combining the spatial supply situation, the use of trace density can more accurately reflect animal habitat selection preferences, which is more in line with the OFT and animal habits. This study reveals the spatial relationship between animal behavior and habitat, which can provide for animal spatial selection research and spatial management decision‐making in reserves and enhance the scientific rigor of ecological studies and the effectiveness of habitat restoration. Therefore, it is recommended to strengthen the control of anthropogenic disturbance (such as grazing) in the gentle slope areas of the reserves in the future, while also paying attention to the population of sympatric animals such as *Budorcas taxicolor bedfordi* etc., to avoid overrunning and occupying suitable space for giant pandas on a large scale, and to increase the availability of gentle slopes so that wild giant pandas can find preferred habitats.

## Author Contributions

**Chun Yang:** formal analysis (equal), investigation (equal), methodology (equal), visualization (equal), writing – original draft (equal). **Yuhang Wang:** investigation (equal), project administration (equal), validation (equal). **Wanyu Wen:** investigation (equal), supervision (equal), validation (equal). **Zhijiao Yang:** data curation (equal), validation (equal), writing – review and editing (equal). **Changcai Li:** data curation (equal), writing – review and editing (equal). **Simeng Yang:** investigation (equal), validation (equal), writing – review and editing (equal). **Xinyu Li:** data curation (equal), writing – review and editing (equal). **Minghao Gong:** conceptualization (equal), funding acquisition (equal), methodology (equal), project administration (equal).

## Conflicts of Interest

The authors declare no conflicts of interest.

## Supporting information


Data S1

## Data Availability

The authors confirm that the data supporting the findings of this study are available within the article and its Supporting Information.
